# Does video assistant referee technology change the magnitude and direction of home advantages and referee bias? A proof-of-concept study

**DOI:** 10.1186/s13102-024-00813-9

**Published:** 2024-01-18

**Authors:** Ali Işın, Qing Yi

**Affiliations:** 1 Department of Coaching Education, Faculty of Sport Sciences, Akdeniz University, Antalya, Turkey; 2 College of Physical Education, Dalian University, 116622 Dalian, China

**Keywords:** Referee decision, Video-replay technology, Decision making, Performance analysis, Match analysis

## Abstract

**Background:**

This study analyzed how Video Assistant Referee (VAR), introduced to improve the accuracy of referee decisions in football, changes the magnitude and direction of home advantage and referee bias in the Turkish Super League.

**Methods:**

We analyzed points, goals, yellow cards and red cards, fouls, penalties, and offside data from 1,838 matches played in the Turkish Super League with and without VAR. Two-sample t-tests and two one-sided tests analysis were applied to determine the differences between the home and away team data between the seasons played with and without VAR.

**Results:**

The findings revealed that the only variable that changed significantly after VAR was implemented was fouls, which decreased for both home (*p* <.001; d = 0.56, medium effect) and away teams (*p* <.001; d = 0.69, medium effect). The results also indicated that, with or without VAR, home teams had an advantage over away teams in points and goals, and away teams faced more referee bias regarding yellow cards and penalties (against).

**Conclusions:**

Although this study shows that VAR does not significantly impact the HA and referee bias of football matches, nevertheless, teams should be more aware that bias is reduced when playing away. In addition, this study offers some practical applications that can help football players, coaches, and match officials better understand VAR technology’s effects on HA and referee bias.

## Introduction

Football (i.e., soccer) is a global sport that attracts millions of fans and players, but it also undergoes constant changes and innovations to meet new demands and opportunities [1]. Technology is a key factor in improving the quality, safety, and entertainment of sports, both on and off the pitch [2]. Some of the technological tools used in football include additional assistant referee [3], goal line technology [4] and vanishing spray [5]. Among these technologies, goal-line technology has substantially improved referee decision-making in football, especially when the ball is not visible due to obstacles [6].

Although these technologies have been used over time, referees’ decisions are still debated. In order to minimize this situation, Video assistant referee (VAR) technology has recently been introduced [7]. VAR is a match official in association football who reviews decisions made by the referee using video replays. The VAR can only intervene in four match-changing situations: goals, penalty decisions, direct red card incidents, and mistaken identity. The VAR contacts the referee through an earpiece and advises the referee to either confirm, change, or review their decision. The referee can check the images on a pitch-side monitor before deciding. The referee always has the final decision to accept or reject the VAR’s advice. The VAR system aims to improve the accuracy and consistency of referee decisions and reduce injustice and controversy [8].

Studies analyzing the impact of VAR on referee decisions [9, 10] and football game [11–13] indicated that VAR implementation leads to more accurate refereeing decisions [9] and a decrease in offsides, fouls, and yellow cards [11]. Spitz et al. (2021) analyzed the impact of VAR on the accuracy of referee decisions, examining the referee’s initial and final decisions after VAR intervention. According to the study the accuracy rate of the referees’ final decisions increased from 92.1 to 98.3% after VAR intervention [9]. However, while there is some evidence that VAR has some impact on football game, Lago-Peñas, Gómez (12) stated that the impact of VAR on elite-level play remains relatively negligible.

In football, the impact on home advantage (HA) and referee bias is considered one of the most debated issues related to this technology [14]. HA is a phenomenon where home teams are believed to have an advantage over away teams due to crowd support and familiarity with the venue. On the other hand, the performance of away teams is affected due to travel fatigue and psychological pressure from home fans. Another phenomenon related to HA is referee bias, which affects referees’ decisions due to crowd noise and social pressure from home fans [15]. Recently, HA has been the topic of research in several sports, particularly football [16, 17]. Several authors have demonstrated the existence of HA and emphasized that home teams have an important advantage [18, 19]. Moreover, HA and referee bias have been demonstrated at different league levels [15, 20] and similarly in women’s football [21]. However, some studies have suggested that technology can reduce HA and referee bias by providing more objective and accurate information [14]. For instance, goal-line technology can prevent errors in goal decisions that could affect the outcome of a match [6].

The Covid-19 outbreak worldwide led to the introduction of VAR and the removal of spectators in many sports competitions [17, 22]. Many researchers have found that HA decreases in ghost matches compared to matches with spectators [16, 17, 20, 23, 24]. However, they did not take into account the possible effect of VAR on HA. Wunderlich et al. (2021) argued that HA persists even without spectators, suggesting that other factors besides the influence of spectators on the team’s performance or the referee’s decision-making contribute to HA [25]. Thus, there is a need for a better understanding of the effects of VAR technology on HA and referee bias. Furthermore, considering the specific characteristics of the leagues, the results of the Turkish league may be different from the others [26]. Moreover, to the best of our knowledge, no studies are currently available that investigate this matter specifically concerning the Turkish Super League. Therefore, this research aimed to check how VAR technology may have an impact on the mean scores of home and away teams in some performance indicators (points, goals, fouls, yellow cards, red cards, offsides and penalties), as well as to analyze the magnitude and direction of the HA and a plausible referee bias in the Turkish Super League.

## Method

### Match sample

This study analyzed 918 matches before VAR was introduced in the 2018–2019 season and 920 matches after VAR was introduced. In the seasons from 2015 to 16 to 2019–2020, 306 matches were played each season. In the 2020–2021 season, 420 matches were played, and in the 2021–2022 season, 380 matches were played. Matches played without fans (n:492) during Covid-19 were excluded from the analysis to control for the possible influence of spectator presence on HA and referee bias.

### Procedure

The study used a retrospective-quantitative and observational (descriptive) study design with two independent groups (seasons with and without VAR) and seven dependent variables (points, goals, fouls, yellow cards, red cards, offsides, and penalties). The study evaluated HA with points and goals, and referee bias with yellow cards, red cards, offsides, and penalties. HA was also computed using Pollard’s method [27], which expresses the proportion of points won at home out of the total points won at home and away [19].The InStat Scout (InStat®), a database with high inter-observer [28] and inter-operator reliability [29], was used to collect the data. This study adhered to the Declaration of Helsinki and received ethical approval from the Akdeniz University Social and Humanities Scientific Research and Publication Ethics Committee (2023-14/325).

### Statistical analysis

The data were analyzed with Jamovi version 2.3.18.0 (The Jamovi project) and each variable’s mean and standard deviation were reported. The normality of the data was tested with the Shapiro-Wilk test (*p* <.05). Two-sample t-tests and two one-sided tests (TOST) analysis [30] were used to compare the groups for each variable. A small effect size (ES) (Cohen’s d ± 0.20) was set as the boundary value for this study. The alternative hypothesis H1: (− 0.20 < d < 0.20) states that the true effect is within the equivalence bounds, meaning that the compared means are practically similar, and it is tested against the composite null hypothesis H0: (d ≤ − 0.20 ∪ d ≥ 0.20) that states that the true effect is large enough to be of interest. If TOST method could reject this null hypothesis, it would be said that the difference between home and away teams was statistically equivalent [31].

The ESs were computed with Cohen’s d and the Bayes factors (BF_01_) were calculated to evaluate the evidence for or against the null hypothesis. The null hypothesis is supported by anecdotal, substantial, strong, very strong and decisive evidence when the Bayes factors (BF_01_) are 1–3, 3–10, 10–30, 30–100, > 100 respectively [32].

## Results

The results indicated that home team outperformed away team on average regarding points and goals variables, which was statistically significant (*p* <.001). However, the ESs were moderate, and the Bayes factors supported the alternative hypothesis of difference (BF01 = 0.00). In addition, away teams faced more yellow cards and penalties (against) than home teams in both seasons, with or without VAR, indicating a possible referee bias. The Table 1 implied that HA and referee bias were present in both groups and that VAR does not have a major impact on the teams’ selected data.

**Table 1:** Comparison of HA and referee bias in matches played with and without VAR

	Variables ^a^	Descriptive statistics				Independent Samples t-test					TOST results for statistical equivalence H0: (d ≤ − 0.20 U d ≥ 0.20) vs. H1: (-0.20 < d < 0.20)		
		Home Team		Away Team									
		M	SD	M	SD	*t*	*p*	ES	BF_01_ (H0/H1) ^b^		Lower bound	Upper bound	Statistical equivalence
*NO VAR* (n: 918)	*Points*	1.65	1.33	1.11	1.28	8.92	< 0.001	0.42	0.00	-	t = 5.63 ^ns^	t = 12.21 ^***^	
	*Goals*	1.58	1.30	1.21	1.17	6.48	< 0.001	0.30	0.00	-	t = 3.01 ^ns^	t = 9.951 ^***^	
	*Fouls*	15.64	4.24	15.63	4.41	0.05	0.961	0.00	19.04	Strong	t = -0.94 ^ns^	t = 1.04 ^ns^	
	*Yellow cards*	2.18	1.36	2.35	1.40	-2.63	0.009	-0.12	0.62	-	t = -5.74 ^***^	t = 0.48 ^ns^	
	*Red cards*	0.12	0.34	0.15	0.42	-1.40	0.162	-0.07	7.24	Substantial	t = -12.56 ^***^	t = 9.76 ^***^	X
	*Offsides*	2.09	1.61	1.92	1.67	2.29	0.022	0.11	1.41	Anecdotal	t = -0.32 ^ns^	t = 4.90 ^***^	
	*Penalties*	0.20	0.44	0.14	0.36	3.36	< 0.001	0.16	0.07	-	t = -7.27 ^***^	t = 13.98 ^***^	X
*VAR* (n: 920)	*Points*	1.63	1.31	1.11	1.25	8.70	< 0.001	0.41	0.00	-	t = 5.34 ^ns^	t = 12.05 ^***^	
	*Goals*	1.58	1.28	1.19	1.12	7.04	< 0.001	0.33	0.00	-	t = 3.47 ^ns^	t = 10.61 ^***^	
	*Fouls*	13.36	3.81	12.80	3.75	3.21	0.001	0.15	0.12	-	t = 2.07 ^ns^	t = 4.34 ^***^	
	*Yellow cards*	2.11	1.37	2.25	1.39	-2.13	0.033	-0.10	2.02	Anecdotal	t = -5.24 ^***^	t = 0.98 ^ns^	
	*Red cards*	0.12	0.36	0.15	0.039	-1.31	0.191	-0.06	8.16	Substantial	t = -12.78 ^***^	t = 10.16 ^***^	X
	*Offsides*	1.99	1.62	1.86	1.52	1.81	0.071	0.08	3.78	Substantial	t = -0.92 ^ns^	t = 4.53 ^***^	
	*Penalties*	0.23	0.49	0.17	0.40	3.14	0.002	0.15	0.14	-	t = -6.50 ^***^	t = 12.78 ^***^	X

^a^ All data presented on a per-match basis

^b^ Computed considering a one-sided null hypothesis

M: Mean, SD: Standard Deviation, *ES*: Cohen’s d effect size, BF_01_: Bayes Factor

*P*-values legend: *p <.05; **p <.01, ***p <.001; ns: not significant (*p* >.05)

Table 2 compares the home team’s data in the seasons before and after the introduction of VAR. It was stated that only one variable (fouls) has a statistically significant difference between the two groups (*p* <.001) for both the t-test and TOST. The ES for this variable was 0.56, which is considered a medium effect. The Bayes factor for this variable was 0.00, indicating strong evidence for the alternative hypothesis. There was no statistical equivalence between the means of this variable. For all other variables, there was no statistically significant or equivalent difference between the two groups (*p* >.05) for both the t-test and TOST. The ESs for these variables were very small (0.00 to 0.06), indicating negligible differences between the groups. The Bayes factors for these variables were very large (7.51 to 19.02), indicating substantial to strong evidence for the null hypothesis.

**Table 2:** Comparison of home team’s data in the seasons before and after the introduction of VAR

Variables ^a^	Descriptive statistics				Independent Samples t-test					TOST results for statistical equivalence H0: (d ≤ − 0.20 U d ≥ 0.20) vs. H1: (-0.20 < d < 0.20)		
	NO VAR (n: 918)		VAR (n: 920)									
	M	SD	M	SD	*t*	*p*	ES	BF_01_ (H0/H1) ^b^		Lower bound	Upper bound	Statistical equivalence
*Points*	1.65	1.33	1.63	1.31	0.47	0.641	0.02	17.13	Strong	t = -2.79^**^	t = 3.72 ^***^	X
*Goals*	1.58	1.30	1.58	1.28	0.02	0.983	0.00	19.07	Strong	t = -3.31^***^	t = 3.35 ^***^	X
*Fouls*	15.64	4.24	13.36	3.81	12.10	< 0.001	0.56	0.00	-	t = 11.03 ^ns^	t = 13.16 ^***^	
*Yellow cards*	2.18	1.36	2.11	1.37	1.07	0.286	0.05	10.86	Strong	t = -2.08^*^	t = 4.21 ^***^	X
*Red cards*	0.12	0.34	0.12	0.36	0.02	0.987	0.00	19.07	Strong	t = -12.21^***^	t = 12.24 ^***^	X
*Offsides*	2.09	1.61	1.99	1.62	1.37	0.170	0.06	7.51	Substantial	t = -1.28 ^ns^	t = 4.02 ^***^	
*Penalties*	0.20	0.44	0.23	0.49	-1.28	0.199	-0.06	8.41	Substantial	t = -10.52^***^	t = 7.95 ^***^	X

^a^ All data presented on a per-match basis

^b^ Computed considering a one-sided null hypothesis

M: Mean, SD: Standard Deviation, *ES*: Cohen’s d effect size, BF_01_: Bayes Factor

*P*-values legend: *p <.05; **p <.01, ***p <.001; ns: not significant (*p* >.05)

Table 3 compares the data of away teams in the seasons with and without VAR. Fouls were the only variable that showed a statistically significant difference between the groups (*p* <.001) for both the t-test and TOST. This variable had a large ES of 0.69 and a Bayes factor of 0.00, which supported the alternative hypothesis. However, the means of this variable were not statistically equivalent. None of the other variables had a statistically significant or equivalent difference between the groups (*p* >.05) for the t-test and TOST. These variables had very small ESs (0.00 to 0.07) and large Bayes factors (5.95 to 19.02), which favored the null hypothesis.

**Table 3:** Comparison of away team’s data in the seasons before and after the introduction of VAR

Variables ^a^	Descriptive statistics				Independent Samples t-test					TOST results for statistical equivalence H0: (d ≤ − 0.20 U d ≥ 0.20) vs. H1: (-0.20 < d < 0.20)		
	NO VAR (n: 918)		VAR (n: 920)									
	M	SD	M	SD	*t*	*p*	ES	BF_01_ (H0/H1) ^b^		Lower bound	Upper bound	Statistical equivalence
*Points*	1.11	1.28	1.11	1.25	0.08	0.938	0.00	19.02	Strong	t = -3.31^***^	t = 3.47 ^***^	X
*Goals*	1.21	1.17	1.19	1.12	0.42	0.678	0.02	17.51	Strong	t = -3.33^***^	t = 4.16 ^***^	X
*Fouls*	15.63	4.41	12.80	3.75	14.82	< 0.001	0.69	0.00	-	t = 13.77 ^ns^	t = 15.87 ^***^	
*Yellow cards*	2.35	1.40	2.25	1.39	1.53	0.125	0.07	5.95	Substantial	t = -1.54 ^ns^	t = 4.61 ^***^	
*Red cards*	0.15	0.42	0.15	0.039	0.13	0.895	0.01	18.91	Strong	t = -10.44^***^	t = 10.71 ^***^	X
*Offsides*	1.92	1.67	1.86	1.52	0.81	0.415	0.04	13.72	Strong	t = -1.87^*^	t = 3.50 ^***^	X
*Penalties*	0.14	0.36	0.17	0.40	-1.45	0.147	-0.07	6.73	Substantial	t = -12.69^***^	t = 9.79 ^***^	X

^a^ All data presented on a per-match basis

^b^ Computed considering a one-sided null hypothesis

M: Mean, SD: Standard Deviation, *ES*: Cohen’s d effect size, BF_01_: Bayes Factor

*P*-values legend: *p <.05; **p <.01, ***p <.001; ns: not significant (*p* >.05)

The introduction of VAR does not significantly impact HA, as the seasons with (59.8%) and without VAR (59.5%) have comparable HA values.

## Discussion

The VAR system was introduced to improve the accuracy and consistency of referee decisions and reduce injustice and controversy. It has been gradually introduced in many leagues since the 2017-18 season and used in the Turkish Super League since 2018–2019 [10]. Despite the numerous studies on how VAR affects match performance, the impact of VAR on HA remains largely unexplored. This research explored how VAR affected HA and referee bias in football seasons and found that VAR did not significantly change these phenomena. This study also analyzed the differences between home and away teams in seasons with and without VAR. The results showed that fouls were the only variable significantly decreasing for both teams after VAR was introduced. The groups had no significant or equivalent difference for all other variables, such as goals, shots, cards, etc.

The first research to comprehensively focus on HA and referee bias in the Turkish Super League pointed to the home advantage in the Super League [26]. Similarly, Işın and Gómez Ruano (24) emphasized that HA exists in the Turkish Super League but is reduced in matches played behind closed doors (ghost matches), thus highlighting the importance of the role of the 12th man. More recently, Işın (15) underlined that HA at different league levels in Turkish football continues regardless of the league level. These results reveal that HA has existed in Turkish leagues for many years and playing at home has a significant advantage.

The impact of VAR on home-field advantage is hard to estimate beforehand. One possibility is that VAR could reduce the tendency of referees to favor the home team in crucial match events and thus make the game fairer. Even though referee bias is not explicitly stated, the main aim of VAR was to “decrease injustice” in referee decisions, implying that bias may be a hidden motive for VAR implementation [33]. This study found that home teams had significantly more points and goals than away teams before and after VAR’s introduction. This means that the HA that existed before VAR was not reduced by it and suggests that VAR is not effective in lowering HA. This study also found that home teams got fewer yellow cards and more penalties than away teams with or without VAR. This indicates some referee bias in favor of home teams in the seasons without VAR, which continued in the seasons with VAR. With the introduction of VAR, both home and away teams had fewer yellow cards and more penalties, but the number of red cards stayed the same. The main reason for this result is that the referees’ independent judgment could explain what appeared to be referee bias in the Turkish Super League in the seasons without VAR. Because after the introduction of VAR, all referee decisions were affected in a similar way and direction for both teams. Using data from 16 leagues, Abbate, Cross (33) examined how VAR systems affect HA in football. They found that VAR had a negligible impact on HA, even though it changed some match statistics for both teams. They argued that VAR only covers a few events that can influence match outcomes and that the observed differences between teams in goals, wins and yellow cards may reflect the effect of fans on players rather than the referee. Unlike these findings, a weak association was found between the introduction of VAR and the reduction of HA in the Chinese Super League. However, the authors argue that VAR may still have some effect on mitigating HA and referee bias [34]. This is because home matches [35] and crowd noise can sway the referees’ judgment [18] and make them favor the home team more often [36]. Using data from 2448 matches played in the four seasons before and after the introduction of VAR, Dufner, Schütz (14) examined the presence of HA in terms of match outcomes and referee bias indicators. HA was evident in points won, goals scored, and yellow cards given before VAR was introduced but not after VAR was implemented for any of the indicators.

The introduction of VAR was associated with reduced fouls and offsides for both teams, as shown in this and previous research [14, 34]. This may be because VAR allows players to see the referees’ decisions on the screen and allows referees to change their decisions after watching the video. Knowing that referees can review their mistakes with the help of VAR, players may have been more careful to avoid fouls [11] and offsides. In addition, assistant referees are cautious when making an offside decision. They avoid making a decision that is not 100% certain, as they know that VAR will review the play to determine whether it is truly offside.

Some limitations to this study should be considered. As a results of studies on the impact of VAR on referee bias are inconsistent across leagues, this may depend on factors such as referee training, home crowd size, weather, and environmental conditions, which vary from league to league. The current study only examined the effects of VAR on HA and referee bias in the Turkish Super League, which may not reflect the general impact of VAR on football. Therefore, future research trends should explore VAR’s HA and referee bias by focusing on data from multiple tournaments or leagues. Future research could also investigate the effects of VAR on home advantage and referee bias, using a different approach such as the mixed model. Besides that, it would be interesting to analyze other contextual variables such as team quality, as well as the type of competition. Finally, this study did not control for some aspects, such as psychological and other performance indicators. Therefore, there is a need to consider psychological and other performance indicators in future studies. Additionally, coaches and players should be analyzed for their opinions on the implementation and its effects on performance.

## Conclusion

This study analyzed the effect of VAR on HA and referee bias in football. The findings of our study showed that, with or without VAR, home teams had an advantage over away teams in points and goals, and away teams faced more referee bias regarding yellow cards and penalties (against). We also found that referees favored home teams in the seasons without VAR, and this favoritism continued in the seasons with VAR. Yet, referees’ independent decisions rather than external pressure can explain this referee bias in the Turkish Super League, as a similar effect continued after VAR.

In conclusion, the VAR technology only allows the referee to correct the referee’s decision for clear and obvious errors. This implies that, during the match, the referee’s discretion in making minor decisions might favor the home team and contribute to their home advantage. Therefore, rather than reducing the HA, VAR may correct critical errors in favor of the away team. As a result, the VAR technology is unlikely to have a significant effect in reducing HA in the Turkish Super League.

## Data Availability

No datasets were generated or analysed during the current study.
